# Supplementary material for: Polyketide synthase gene clusters in the ericoid mycorrhizal fungus Oidiodendron maius: genome-wide prediction, expression profiling, and functional characterization of knockout mutants
Source: Front Plant Sci. 2026 Sep 16;17:1924430. doi: 10.3389/fpls.2026.1924430 (PMC13623903; doi:10.3389/fpls.2026.1924430)
Supplement: Supplementary file 1 [file SupplementaryFile1.zip › Supplementary Materials/Supplementary Folder 1.zip!/Oidma1_AssemblyScaffolds_Repeatmasked/knownclusterblast/cluster55/jgi.p_Oidma1_21637_mibig_hits.html]

| MIBiG Protein | Description | MIBiG Cluster | MiBiG Product | % ID | % Coverage | BLAST Score | E-value |
| --- | --- | --- | --- | --- | --- | --- | --- |
| EAL89347.1 | hypothetical\_protein | BGC0001403 | Trypacidin\_biosynthetic\_gene\_cluster | 79 | 88.1578947368 | 242 | 4.4e-64 |
| ADM34150.1 | hypothetical\_protein | BGC0001084 | Notoamide\_/\_stephacidin\_biosynthetic\_gene\_cluster | 73 | 95.3947368421 | 235 | 5.4e-62 |
| CBF90095.1 | conserved\_hypothetical\_protein | BGC0001271 | Emericellin\_biosynthetic\_gene\_cluster | 62 | 96.7105263158 | 197 | 9.5e-51 |
| AGO59044.1 | PtaD | BGC0000121 | Pestheic\_acid\_biosynthetic\_gene\_cluster | 61 | 88.8157894737 | 178 | 7.8e-45 |
